# Sex difference in cerebrospinal fluid/blood albumin quotients in patients with schizophreniform and affective psychosis

**DOI:** 10.1186/s12987-020-00223-2

**Published:** 2020-11-11

**Authors:** Sophie Meixensberger, Karl Bechter, Rick Dersch, Bernd Feige, Simon Maier, Miriam A. Schiele, Kimon Runge, Dominik Denzel, Kathrin Nickel, Derek Spieler, Horst Urbach, Harald Prüss, Katharina Domschke, Ludger Tebartz van Elst, Dominique Endres

**Affiliations:** 1 Section for Experimental Neuropsychiatry, Department of Psychiatry and Psychotherapy, Medical Center-University of Freiburg, Faculty of Medicine, University of Freiburg, Freiburg, Germany; 2 Department of Psychiatry and Psychotherapy, Medical Center-University of Freiburg, Faculty of Medicine, University of Freiburg, Freiburg, Germany; 3 Department for Psychiatry and Psychotherapy II, Ulm University, Bezirkskrankenhaus Günzburg, Günzburg, Germany; 4 Department of Neurology, Medical Center-University of Freiburg, Faculty of Medicine, University of Freiburg, Freiburg, Germany; 5 Department of Psychosomatic Medicine and Psychotherapy, Medical Center-University of Freiburg, Faculty of Medicine, University of Freiburg, Freiburg, Germany; 6 Department of Neuroradiology, Medical Center-University of Freiburg, Faculty of Medicine, University of Freiburg, Freiburg, Germany; 7 Department of Neurology and Experimental Neurology, Charité-Universitätsmedizin Berlin, Berlin, Germany; 8 German Center for Neurodegenerative Diseases (DZNE), Berlin, Germany; 9 Center for Basics in Neuromodulation, Faculty of Medicine, University of Freiburg, Freiburg, Germany

**Keywords:** Cerebrospinal fluid, Albumin quotient, Protein, Psychosis, Sex

## Abstract

**Background:**

The importance of cerebrospinal fluid (CSF) diagnostics for psychiatry is growing. The CSF/blood albumin quotient (Q_Alb_) is considered to be a measure of the blood–CSF barrier function. Recently, systematically higher Q_Alb_ in males than in females was described in neurological patients. The aim of this study was to investigate whether a sex difference could also be detected in a well-characterized psychiatric cohort.

**Methods:**

The patient cohort comprised 989 patients, including 545 females and 444 males with schizophreniform and affective syndromes who underwent CSF diagnostics, including Q_Alb_ measurement. The basic CSF findings and antineuronal autoantibody data of this cohort have already been published. This re-analysis employed analysis of covariance with age correction for Q_Alb_ mean values and chi^2^-testing for the number of increased age-corrected Q_Alb_ levels to investigate sex differences in Q_Alb_.

**Results:**

The Q_Alb_ levels were elevated above reference levels by 18% across all patients, and a comparison between male and female patients revealed a statistically significant sex difference, with increased values in 26% of male patients and a corresponding rate of only 10% in female patients (*chi*^2^ = 42.625, *p* < 0.001). The mean Q_Alb_ values were also significantly higher in males (6.52 ± 3.69 × 10^–3^) than in females (5.23 ± 2.56 × 10^–3^; *F* = 52.837, *p* < 0.001).

**Discussion:**

The main finding of this study was a significantly higher Q_Alb_ level in male compared to female patients with psychiatric disorders, complementing previously described sex differences in neurological patient cohorts. This result indicates bias from some general factors associated with sex and could be partly explained by sex differences in body height, which is associated with spine length and thus a longer distance for CSF flow within the subarachnoid space down the spine from the occipital area to the lumbar puncture site in males compared to females. Hormonal influences caused by different estrogen levels and other sex-specific factors could also play a relevant role. The significance of the study is limited by its retrospective design, absence of a healthy control group, and unavailability of exact measures of spine length.

## Background

Interest in the immunological pathways underlying the etiology and pathophysiology of a subgroup of patients with mental disorders has increased in recent years. The cerebrospinal fluid (CSF) is a dynamic, metabolically active secretion that can provide important information pertaining to inflammatory changes involving the brain [1]. Therefore, diagnostic examinations of CSF composition have been recommended increasingly in the study of severe mental disorders [2]. The blood–brain barrier (BBB) is the primary interface dividing peripheral circulation from the central nervous system (CNS) [3]. Its most notable component is the cerebral microvascular endothelium, which functions as a diffusion barrier with selective uptake and efflux transporters and specific vesicular transport [3–6]. Endothelial tight junctions restrict the paracellular pathway between the blood and brain for polar solutes [5, 7]. The CSF/blood albumin quotient (Q_Alb_) has been considered the gold standard for assessment of the BBB function [7]. However, a complicated system of three or more brain barriers and interfaces is now recognized [8–11]. Therefore, Q_Alb_ levels are now considered a measure of the overall blood-CSF-barrier (BCSFB) [12, 13] in the framework of the flow-diffusion model of BCSFB (dys-)function originally proposed by Reiber [13–15]. Thus, the overall BCSFB is a construct created to explain the complicated interrelationship between CSF and surrounding tissues and the many dynamic influences of exchange involved, as most recently seen in analysis of CSF composition for clinical purposes. Albumin is synthesized predominantly in the liver, from which it rapidly enters the bloodstream and enters CSF circulation mainly during the process of CSF production by the choroid plexus in the brain ventricles, with albumin representing approximately 80% of normal CSF total protein [12]. However, recent research results also demonstrated that under certain circumstances albumin can also be produced in the brain by microglial cells [16, 17]. With increasing age, the permeability of the BBB/BCSFB increases, whereas CSF production and thus the CSF turnover rate declines [12, 13]. Both of these factors contribute to a distinct age-dependent increase of Q_Alb_; thus, Q_Alb_ reference values are generally corrected for age [12, 13]. Moreover, Q_Alb_ could be influenced by other factors such as body weight, body mass index, a reduction in CSF production from reasons other than age, alcohol and nicotine consumption [1, 14]. Interestingly, an influence of sex on Q_Alb_ was recently also noticed and discussed in two papers that investigated patient cohorts with a spectrum of neurological disorders [18, 19].

The aim of this study was to investigate whether similar sex differences in Q_Alb_ can be detected in a cohort of psychiatric patients by reanalyzing the data of a recently published study of a well-characterized sample of patients with schizophreniform and affective psychosis [20].

### Methods

The retrospective data analyses received approval from the local ethics committee of the University of Freiburg (EK Fr 396/18). Between January 2006 and November 2019, 992 patients were investigated [20], of which only those patients who had been tested for Q_Alb_ were analyzed in the current study (N = 989).

### Female and male patient groups

All patients received a lumbar puncture (LP) for the organic clarification of their mental disorder. Schizophreniform syndromes (according to the International Statistical Classification of Diseases and Related Health Problems criteria, version 10 [ICD-10]: F20.X–F29.X, F06.0–2, F10.5–F19.5) and affective syndromes (unipolar depression following ICD-10: F32.X, F33.X, F06.3; bipolar disorder following ICD-10: F30.X, F31X, F06.3) were clinically diagnosed by experienced psychiatrists and classified according to their predominant initial psychiatric syndromes. Preexisting or newly occurring neurological comorbidities were itemized but not considered as exclusion criteria whereas a comorbid diagnosis of dementia (ICD-10: F00.X–F04.X) led to exclusion. The clinical characteristics were extracted from the patients’ discharge letters. The clinical findings and psychometric scores, including Clinical Global Impression (CGI), Global Assessment of Functioning (GAF), and psychopathological scores following the German Association for Methodology and Documentation in Psychiatry (AMDP), were extracted from the basic clinical documentation.

### Laboratory methods

Paired CSF and serum samples were drawn at the same time following guideline recommendations [21]. The CSF and serum diagnostics were performed in the CSF laboratory of the Department of Neurology of the University Medical Center Freiburg (https://www.uniklinik-freiburg.de/neurologie/klinik/diagnostische-einrichtungen/liquor-labor.html), and the CSF and serum samples were analyzed immediately after centrifugation. The basic quantitative protein diagnostics via nephelometry included total CSF protein concentration, serum/CSF albumin, and serum/CSF IgG concentration (ProSpect System, Siemens, Erlangen, Germany). The CSF and serum albumin were used to calculate the Q_Alb_ using the formula Q_Alb_ = [Alb]CSF/[Alb]serum × 1000. The established age-dependent reference values for Q_Alb_ were used: < 40 years: < 6.5 × 10^–3^; 40–60 years: < 8 × 10^–3^; and > 60 years: < 9.3 × 10^–3^ [22]. In addition, the white blood cell (WBC) count and cytological staining were assessed by manual microscopy (Leica DMRB, Germany) using a Fuchs-Rosenthal counting chamber (Hecht-Assistant, Germany). All samples with elevated WBC counts were checked for blood contamination. In case of blood contamination the WBC counts were corrected according to the following formula: 1 cell/µl of WBC count reduction per 1000 red blood cells/µl. Isoelectric focusing followed by immunofixation (Hydragel Isofocusing, Sebia, France) were used to determine oligoclonal bands (OCBs).

### Instrument-based diagnostics

#### Electroencephalography (EEG)

The admission procedures included an EEG examination in resting state and under hyperventilation (HV). The evaluation was performed as part of clinical routine [20].

#### Magnetic resonance imaging (MRI)

At the least, the MRI protocol included T1-weighted (MPRAGE with isotropic 1 mm^3^ voxels on 3 T or axial 5 mm thick fast spin-echo slices on a 1.5 T scanner), FLAIR (3D SPACE sequence with isotropic 1 mm^3^ voxels on 3 T or coronal 3 mm thick fast spin-echo slices on a 1.5 T scanner), and DWI sequences (axial 5 mm thick slices). The evaluation was performed by experienced senior physicians in neuroradiology, and the MRI findings were classified as in the main study [20].

### Available data sets

The initial study cohort included 992 patients [20], of which the Q_Alb_ of 989 patients was available. Those 989 patients were analyzed in the current study. Due to the retrospective approach, some parameters are missing. The available data sets are presented in Table 1.

**Table 1:** Overview of examined parameters of patients

	Parameters	Total N (female/male)
Cerebrospinal fluid (CSF) markers		
Signs of BCSFB dysfunction	Albumin quotients (AQ)	989 (545/444)
Further CSF basic analyses	Total protein	988 (544/444)
	White blood cell count	979 (536/443)
	IgG index	989 (545/444)
	OCBs in CSF	965 (529/436)
	OCBs in serum	964 (529/435)
Instrument-based diagnostics		
EEG	Resting state	951 (519/432)
	Hyperventilation period	802 (441/361)
MRI of the brain	T1/MPRAGE/DWI/FLAIR	894 (498/396)

CSF, cerebrospinal fluid; BCSFB, blood-cerebrospinal fluid barrier; AQ, albumin quotient; IgG, immunoglobulin; OCB, oligoclonal bands; EEG, electroencephalography; MRI, magnetic resonance imaging

### Statistical analyses

**Fig. 1:**
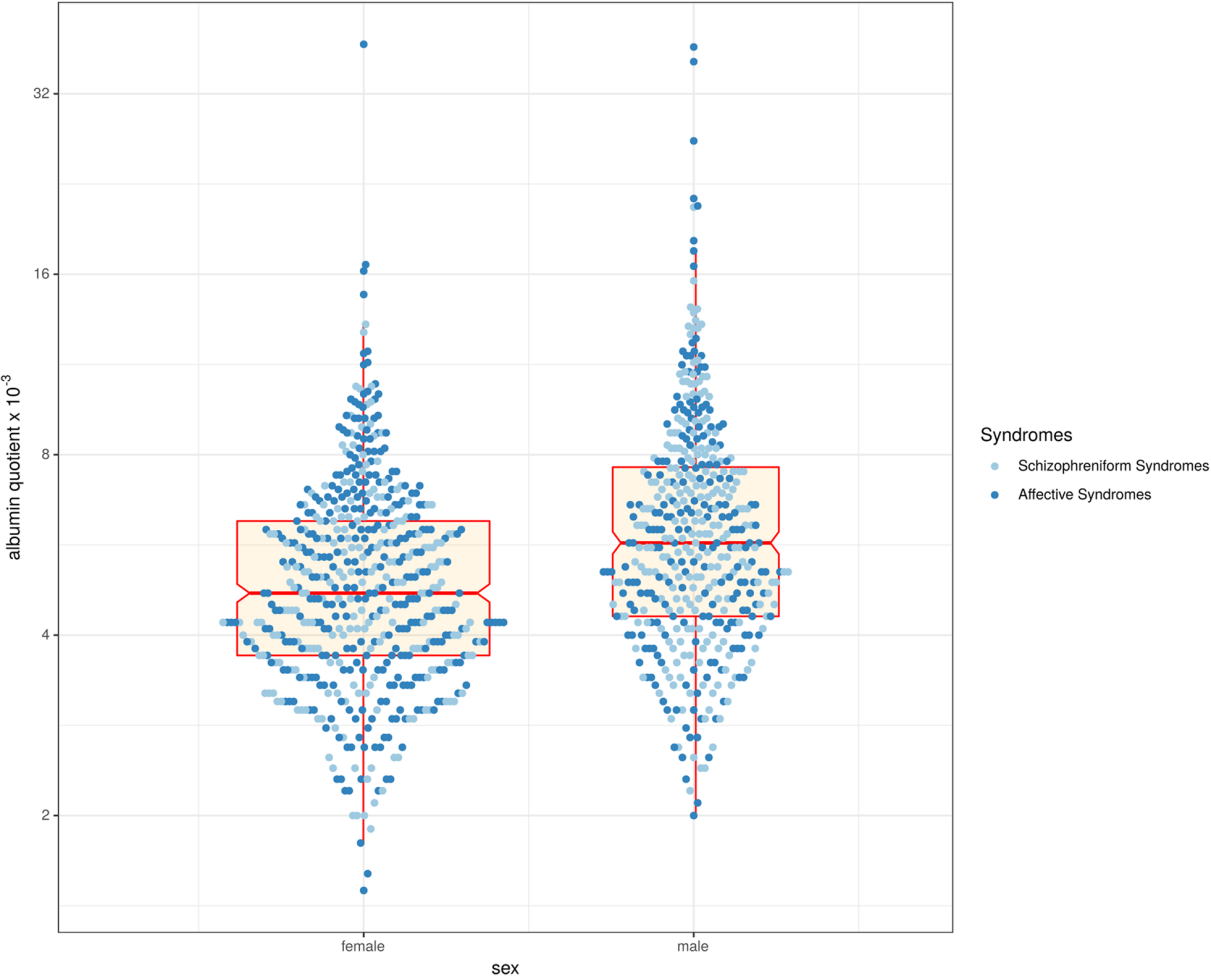
Scatter plot diagram with levels of albumin quotients (y-axis) and sex (x-axis). Single case data is shown. A box plot is added in red (quartiles and median of the distribution; the notches in the box sides give an approximation of the 95% confidence interval around the median). The data are presented on logarithmic scales

Statistical analysis was carried out using the Statistical Package for the Social Sciences (SPSS), version 24 (IBM Corp., Armonk, NY, USA). The results are mainly presented in a descriptive manner. Independent samples t-tests were conducted comparing age and dimensional variables between the subgroups of patients (without age difference). ANCOVA analyses were used to compare other dimensional variables (e.g., CSF Q_Alb_ between female and male patients) between the subgroups corrected for age where indicated. The age-corrected Q_Alb_ values were compared using chi^2^ tests. A binary logistic regression (“Wald statistics”) was performed for age-dependent categorical variables (e.g., the number of elevated CSF protein levels) between groups with age differences. The correlations between Q_Alb_ levels and IRDA/IRTA rates, clinical findings (number of suicide attempts and number of earlier inpatient stays), and psychometric scores (GAF, CGI, and AMDP) were analyzed using Spearman correlation. The correlation analyses were performed separately for female and male patients. A p-value of < 0.05 was defined as statistically significant for Q_Alb_ comparisons (the primary outcome parameter). This significance level was also used for further group comparisons and correlation analyses. Due to the exploratory approach of the additional statistical analyses, no correction for multiple testing was performed. Figure 1 was created using R [23].

## Results

### Description of the study population

The CSF of 545 female and 444 male patients was analyzed. The two subgroups differed significantly in age (females: 43.77 ± 17.21 years, males: 41.33 ± 18.61 years; p = 0.033). Detailed characterizations are presented in Table 2, and medications at the time of LP or upon admission are listed in Table 3.

**Table 2:** Description of the study population

	All patients (N = 989)		Statistics
	Females (N = 545; 55%)	Males (N = 444; 45%)	
Age (range) in years ± SD	43.77 ± 17.21 (from 18–90)	41.33 ± 18.61 (from 18–85)	F = 8.183 p = 0.033
Syndrome			
Schizophreniform syndrome	N = 248 (46%)	N = 208 (47%)	
Affective syndrome			
Depressive syndrome	N = 249 (46%)	N = 203 (46%)	
Bipolar syndrome	N = 48 (9%)	N = 33 (7%)	
Clinical course			
First episode	143 (27%)	135 (30%)	
Chronic (> 2 years)	133 (25%)	125 (28%)	
Recurrent	261 (49%)	183 (41%)	
Unknown	8 (1%)	1 (0.2%)	
Previous/current comorbid psychiatric disorders			
Neurodevelopmental disorders^a^	38 (7%)	51 (11%)	
Personality disorders	26 (5%)	17 (4%)	
Substance abuse/dependence	50 (9%)	63 (14%)	
Anxiety disorders	17 (3%)	16 (4%)	
OCD	17 (3%)	7 (2%)	
PTSD	12 (2%)	6 (1%)	
Cognitive disorders (MCI)	15 (3%)	26 (6%)	
Sleep disturbances	9 (2%)	9 (2%)	
Eating disorders	16 (3%)	0 (0%)	
Somatoform disorder^b^	20 (4%)	12 (3%)	
Others^c^	7 (1%)	2 (0.5%)	
Previous/current comorbid neurological disorders			
Neurovascular	10 (2%)	14 (3%)	
Demyelinating^d^	2 (0.4%)	1 (0.2%)	
Extrapyramidal/movement	3 (0.6%)	7 (2%)	
Infectious	3 (0.6%)	3 (0.7%)	
Tumors	2 (0.4%)	1 (0.2%)	
Paroxysmal	12 (2%)	7 (2%)	
Traumatic injuries	11 (2%)	12 (3%)	
Polyneuropathy	7 (1%)	13 (3%)	
Migraine and other headaches	36 (7%)	10 (2%)	
Restless Legs Syndrome	9 (2%)	7 (2%)	
Hydrocephalus	4 (0.7%)	5 (1%)	
Others	12 (2%)	10 (2%)	
School education			
No degree	9 (2%)	5 (1%)	
Low degree	87 (20%)	86 (23%)	
Medium degree	137 (31%)	97 (26%)	
High degree	201 (46%)	182 (49%)	
Other	3 (0.7%)	5 (1%)	
Unknown	108 (20%)	69 (16%)	
Occupation			
Employed	150 (33%)	114 (30%)	
House-wife/-husband	28 (6%)	0 (0%)	
Unemployed	59 (13%)	67 (18%)	
Disability pension	56 (12%)	45 (12%)	
Retirement pension	72 (16%)	59 (16%)	
In-training/in studies/retraining	68 (15%)	81 (21%)	
Others	18 (4%)	10 (3%)	
Unknown	94 (17%)	67 (15%)	

SD, standard deviation; ADHD, attention deficit hyperactivity disorder; PTSD, post-traumatic stress disorder; OCD, obsessive–compulsive disorder; MCI, mild cognitive impairment

^a^Neurodevelopmental disorders include ADHD, autism, tic disorder; ^b^Somatoform disorders include somatization disorders, hypochondriac disorders, persistent pain disorders. ^c^Other psychiatric comorbidity: Dissociative disorders. ^d^Relapse of multiple sclerosis with pure psychiatric manifestations. Multiple sclerosis has been initially diagnosed

**Table 3:** Psychopharmacological medication

	All patients (N = 989)		Statistics
	Females (N = 545; 55%)	Males (N = 444; 45%)	
Overall psychopharmacological treatment			
Yes	498 (94%)	415 (95%)	Chi^2^ = 0.811 p = 0.368
No	34 (6%)	22 (5%)	
Unknown	13 (1%)	7 (2%)	
Antidepressants			
Overall	295 (59%)	221 (53%)	
Tricyclic	31 (11%)	28 (13%)	
SSRI, SNRI, NDRI, NARI	261 (88%)	188 (85%)	
MAO inhibitors	3 (1%)	5 (2%)	
Antipsychotics			
Overall	384 (77%)	286 (69%)	
“Typical”	83 (17%)	59 (14%)	
Low-potency	61 (73%)	39 (66%)	
Medium-potency	0 (0%)	0 (0%)	
High-potency	22 (27%)	20 (34%)	
“Atypical”	356 (71%)	263 (63%)	
Mood stabilizers			
Lithium	92 (18%)	57 (14%)	
Anticonvulsants	85 (17%)	58 (14%)	
Benzodiazepines	87 (17%)	58 (14%)	
Number of different medication classes per patient			
Same class/only one drug	174 (35%)	202 (49%)	
Two drugs	189 (38%)	135 (33%)	
Three drugs	97 (19%)	68 (16%)	
Four drugs	35 (7%)	10 (2%)	
Five drugs	3 (0.6%)	0 (0%)	

SSRI, selective-serotonin-reuptake-inhibitor; SNRI, serotonin-noradrenalin-reuptake-inhibitor; NDRI, norepinephrine-dopamine-reuptake-inhibitor; NARI, noradrenalin-reuptake-inhibitor; MAO, monoamine oxidase

### CSF-blood albumin quotients

In total, 18% of all patients exhibited elevated Q_Alb_ levels compared to age adjusted control levels [22]. Comparing male and female patients revealed a statistically significant difference (chi^2^ = 42.625, p < 0.001), with a higher number of elevated age-corrected Q_Alb_ in male patients (26%) than in female patients (10%). The mean values of the Q_Alb_ also differed significantly (F = 52.837, CI = [0.892; 1.676], p < 0.001) between female (M ± SD × 10^–3^ = 5.23 ± 2.56) and male patients (M ± SD × 10^–3^ = 6.52 ± 3.69), as illustrated in Fig. 1. This significant sex difference was found irrespective of clinical syndromes (Table 4).

**Table 4:** Signs of blood-cerebrospinal fluid barrier dysfunction: albumin quotients and numbers for female and male patients in relation to reference values

	Reference	All patient (N = 989)	Females (N = 545; 55%)	Males (N = 444; 45%)	Statistics
All patients					
Albumin quotients (× 10^–3^, Mean ± SD, median and range)		5.81 ± 3.18 5.10 (1.50–38.70)	5.23 ± 2.56 4.70 (1.50–38.70)	6.52 ± 3.69 5.70 (2.00–38.30)	F = 52.837 CI = [0.892;1.676] p < 0.001
Increased albumin quotients, numbers of patient	< 40 y.: < 6.5 × 10^–3^ 40–60 y.: < 8 × 10^3^ > 60 y.: < 9.3 × 10^–3^	↑: 174 (18%) ↔ : 815 (82%)	↑: 57 (10%) ↔ : 488 (90%)	↑: 117 (26%) ↔ : 327 (74%)	Chi^2^ = 42.625 p < 0.001

	Schizophreniform syndrome (N = 456)		Females (N = 248; 54%)	Males (N = 208; 46%)	
Albumin quotients (× 10^–3^, Mean ± SD)		5.64 ± 3.18	5.29 ± 3.02	6.06 ± 3.32	F = 11.906 CI = [0.187;1.354] p = 0.001
Increased albumin quotients, numbers of patients	< 40 y.: < 6.5 × 10^–3^ 40–60 y.: < 8 × 10^–3^ > 60y.: < 9.3 × 10^–3^	↑: 85 (19%) ↔ : 371 (81%)	↑: 33 (13%) ↔ : 215 (87%)	↑: 52 (25%) ↔ : 156 (75%)	Chi^2^ = 10.200 p = 0.001

	Affective syndrome (N = 533)		Females (N = 297, 56%)	Males (N = 236, 44%)	
Albumin quotients (× 10^–3^, Mean ± SD)		5.95 ± 3.19	5.19 ± 2.11	6.92 ± 3.96	F = 27.991 CI = [1.208;2.260] p < 0.001
Increased albumin quotients, numbers of patients	< 40 y.: < 6.5 × 10^–3^ 40-60 y.: < 8 × 10^–3^ > 60y.: < 9.3 × 10^–3^	↑: 89 (17%) ↔ : 444 (83%)	↑: 24 (8%) ↔ : 273 (92%)	↑: 65 (28%) ↔ : 171 (72%)	Chi^2^ = 35.808 p < 0.001

SD, standard deviation; CI, confidence interval; ↑, increased; ↔ , in normal range

### The effect of neurological comorbidity as a confounding factor on sex differences

In order to rule out an effect of neurological comorbidities, a post hoc analysis was performed in the subgroup of patients without neurological comorbidities only. Female (age: M = 42.39 years, SD = 16.42; N = 434) and male (age: M = 38.24 years, SD = 17.26; N = 354) patients without neurological comorbidities (N = 788, 80%) differed significantly in age (F = 1.799, p = 0.001). Significant differences (F = 36.029, p < 0.001) in levels of Q_Alb_ were discerned, with higher rates in male (M ± SD × 10^–3^ = 6.21 ± 3.41) than in female patients (M ± SD × 10^–3^ = 5.18 ± 2.57) in the age-corrected ANCOVA analysis.

### Cerebrospinal fluid basic parameters and instrument-based diagnostics

The CSF protein levels were elevated in male as compared to female patients (β = 0.859, Wald = 39.542, p < 0.001). Overall, male patients exhibited some degree of CSF alteration significantly more frequently than female patients (β = 0.747, Wald = 30.608, p < 0.001). There were no significant sex differences in white blood cell counts, the IgG index, or oligoclonal bands (Table 5). EEG analyses revealed no significant sex differences. Female patients more frequently had inflammatory changes in the MRI (Wald = 4.360, p = 0.037, Table 6).

**Table 5:** Cerebrospinal fluid (CSF) basic parameters

	Reference	All patient (N = 989)		Females (N = 545; 55%)	Males (N = 444; 45%)	Statistics
Cerebrospinal fluid (CSF) basic parameters						
CSF protein concentration (Mean ± SD, range)		470.91 ± 236.45 (from 107 to 2890 mg/l)	423.74 ± 196.84		528.70 ± 266.44	F = 60.366 p < 0.001
Increased CSF protein concentration	< 450 mg/l	↑: 447 (45%) ↔ : 541 (55%) n.a.: 1 (0.1%)	↑: 202 (37%) ↔ : 342 (63%) n.a.: 1 (0.2%)		↑: 245 (55%) ↔ : 199 (45%) n.a.: 0 (0%)	β = 0.859 Wald = 39.542 p < 0.001
WBC counts (Mean ± SD)		1.96 ± 4.85 (from 1 to 101 /µl)	1.99 ± 5.33		1.93 ± 4.20	F = 0.070 p = 0.791
Increased WBC counts	< 5 /µl	↑: 37 (4%) ↔ : 942 (96%) n.a.: 1 (0.1%)	↑: 25 (5%) ↔ : 511 (95%) n.a.: 9 (2%)		↑: 12 (3%) ↔ : 431 (97%) n.a.: 1 (0.2%)	Wald = 2.777 p = 0.096
IgG-Index (Mean ± SD)		0.50 ± 0.10	0.50 ± 0.11		0.50 ± 0.09	F = 0.508 p = 0.476
Increased IgG indices		↑: 19 (2%) ↔ : 970 (98%)	↑: 14 (3%) ↔ : 531 (97%)		↑: 5 (1%) ↔ : 439 (99%)	Wald = 2.367 p = 0.124
Isolated OCB in CSF OCBs in CSF and Serum	negative negative	40 (4%) n.a.: 24 (2%) 52 (5%) n.a.: 25 (3%)	21 (4%) n.a.: 16 (3%) 27 (5%) n.a.: 16 (3%)		19 (4%) n.a.: 8 (2%) 25 (6%) n.a.: 9 (2%)	Wald = 0.095 p = 0.758 Wald = 0.456 p = 0.500
Inflammatory CSF changes^a^		77 / 989 (8%)	48 / 545 (9%)		29 /444 (7%)	Wald = 1.754 p = 0.185
Overall Basic CSF changes^b^		491 / 989 (50%)	232 / 545 (43%)		259 / 444 (58%)	β = 0.747 Wald = 30.608 p < 0.001

WBC, white blood cell; OCBs, oligoclonal bands; CSF, cerebrospinal fluid; SD, standard deviation; ↑, increased; ↔ , in normal range; n.a., not available

^a^Inflammatory CSF changes: WBC counts increased and/or IgG indices increased and/or CSF specific oligoclonal bands. ^b^Overall basic CSF alterations: Inflammatory CSF changes and/or increased albumin quotients and/or increased protein concentrations

**Table 6:** Instrument based diagnostics

	All patients (N = 989)	Females (N = 545; 55%)	Males (N = 444; 45%)	Statistics
EEG Alterations	N = 951 (96%)	N = 519 (95%)	N = 432 (97%)	
Continuous generalized slow activity	34 (4%)	18 (3%)	16 (4%)	Wald = 0.105 p = 0.746
Continuous regional slow activity	6 (0.6%)	1 (0.2%)	5 (1%)	Wald = 2.748 p = 0.097
Intermittent generalized slow activity	161 (17%)	95 (18%)	66 (15%)	Wald = 1.789 p = 0.181
Intermittent regional slow activity	52 (5%)	31 (6%)	21 (5%)	Wald = 0.462 p = 0.497
Epileptic pattern	29 (3%)	15 (3%)	14 (3%)	Wald = 0.003 p = 0.958
EEG overall alterations	240 / 951 (25%)	136 / 519 (26%)	104 / 432 (24%)	Wald = 0.641 p = 0.423
MRI Alterations	N = 894 (90%)	N = 498 (91%)	N = 396 (89%)	
White/Grey matter changes overall^a^	460 / 894 (51%)	269 / 498 (54%)	191 / 396 (48%)	Wald = 0.906 p = 0.341
Non-specific white matter changes	374 (42%)	212 (43%)	162 (41%)	Wald = 0.042 p = 0.837
Grey matter changes of amygdalae, hippocampi, other limbic structures	12 (1%)	8 (2%)	4 (1%)	Wald = 0.400 p = 0.527
Possible/probable/ definite (post-) inflammatory changes	77 (9%)	52 (10%)	25 (6%)	Wald = 4.360 p = 0.037
Atrophic changes^b^	107 (12%)	52 (10%)	55 (14%)	Wald = 3.702 p = 0.054
Macroangiopathic vascular alterations (post-ischemic changes)	33 (4%)	17 (3%)	16 (4%)	Wald = 0.306 p = 0.580
Microhemorrhage	16 (2%)	10 (2%)	6 (2%)	Wald = 0.244 p = 0.621
Cysts	118 (13%)	67 (13%)	51 (13%)	Wald = 0.213 p = 0.644
Tumors	13 (1%)	9 (2%)	4 (1%)	Wald = 0.834 p = 0.361
Anatomical variants and other changes	208 (23%)	118 (24%)	90 (23%)	Wald = 0.156 p = 0.693
Overall MRI changes	639 / 894 (71%)	366 / 498 (73%)	273 / 396 (69%)	Wald = 0.531 p = 0.466

Several EEG and MRI changes were noted, if existing.

EEG, electroencephalography; IRDA/IRTA, intermittent rhythmic generalized delta/theta activity; MRI, magnetic resonance imaging

^a^White/grey matter changes overall: non-specific white matter changes and/or gray matter changes of amygdalae, hippocampi, other limbic structures and/or (post-)inflammatory changes. ^b^Atrophic changes overall: generalized cortical atrophy and/or localized atrophy and/or ventricle enlargement

### Correlation analyses

#### Female patients

The Q_Alb_ correlated significantly with the AMDP scores for fears and compulsions (r = − 0.161, p = 0.001; N = 422), and the AMDP scores for delusions (r = − 0.114, p = 0.019; N = 421).

#### Male patients

The Q_Alb_ were significantly correlated with CGI score on admission (r = 0.168, p = 0.001; N = 388), number of earlier suicide attempts (r = 0.213, p = 0.003; N = 197), and the AMDP scores for fears and compulsions (r = -0.161, p = 0.003; N = 352).

## Discussion

The main result of this study is a significantly elevated Q_Alb_ in men compared to women, which at first glance and in accordance with traditional interpretations suggests that the integrity of the BBB is more often impaired in men than in women with schizophrenic and affective psychosis. However, due to current recognition that there are three or more brain barriers and interfaces [8–11], Q_Alb_ is now considered a measure of the overall BCSFB [12, 13, 15]. Q_Alb_ represents a measure of predominantly blood-derived albumin within the CSF in relation to blood albumin concentration. The CSF albumin content determined from lumbar CSF, can potentially be influenced by factors other than plasma concentration. The exchange of CSF and its content with the tissues surrounding the brain ventricles and subarachnoid spaces could alter the composition. Also, variable CSF flow dynamics passing through the subarachnoid spaces to the lumbar puncture site, which lies further down the spine, appears to have an influence.

### Integration of our results into the context of current studies

This finding is consistent with the results of two samples of patients with neurological disorders, including some healthy controls [18, 19]. In a first large study including 27,263 patients with neurological diseases, elevated Q_Alb_ values were observed more frequently in male patients [19]. However, details about the syndromes, diagnostic findings, and medication were not available. The study also included data from 335 healthy controls (although the definition of “healthy” was not specified), who displayed the same sex differences as well [19]. A similar study including 1209 patients with different neurological disorders also reported similar sex differences [18]. In the present study, these findings could be confirmed in a more homogeneous, well-characterized psychiatric cohort including diagnostic findings and information about potential confounding factors, such as comorbidity or psychopharmacological medication. Another study investigating a large cohort of 1,079,193 participants with unknown characteristics evaluated serum albumin and found sex differences depending on age [24]. In the current study, protein concentrations were also higher in male than in female patients, similar to previous studies [25, 26]. Overall, the sex difference in Q_Alb_ levels appears to be independent of neurological or psychiatric diagnoses, which therefore strongly suggests bias from some general factors associated with sex, which remain to be elucidated.

### Possible explanations for the sex differences in albumin quotients

Sex-related differences in body height, specifically spine length, may constitute a plausible supporting factor for this bias, given that sex appears to be predominantly associated with a systematic difference in average height. Since body height was unfortunately not recorded in the present cohort, the average heights of the German population were used as an approximate comparison. Measures for the German population from a microcensus of 2017 reported a mean height for ages 18–40 of 181 cm for males and of 167 cm for females, and for ages 40–65, 179 cm for males and 166 cm for females, respectively (Statistisches Bundesamt, https://www.destatis.de). Thus, extrapolating from a general German population broadly comparable to our patient group (female mean age 43.77 years; male mean age 41.33 years) indicates a systematic height difference between the sexes by approximately 13.5 cm. This height difference implies an analogous difference of spine length between the sexes, although leg length might represent a confounder. The assumed sex differences in body height of > 10 cm and, as a consequence, in spine length between men and women, would be able to explain sex-related increases of Q_Alb_ in males as compared to females because of the longer CSF flow distance down the spinal cord in men and a comparably shorter CSF flow distance in women because of shorter female spine size in related subarachnoid spaces. This explanatory approach is in line with replicated findings of a rostro-caudal gradient of Q_Alb_ with increasing Q_Alb_ values in CSF taken at sites down the neuraxis from the site of production in the ventricles to the most distal sites [14, 15, 27]. It also agrees in principle with a decrease in Q_Alb_ when CSF was taken in sequential portioned volumes at the lumbar site [15, 28], which led to the general recommendation to always take fixed total volumes of CSF for examination to avoid this volume bias in routine diagnostics [12, 13, 21]. In addition, the suggested explanation matches with the flow-diffusion-model of BCSFB [14, 15]. The slow net flow of CSF down the neuraxis within the subarachnoid spaces is apparently related to and determined by the slow outflow of CSF along spinal nerves [29, 30], first described by Quincke in 1872 but widely forgotten until recently [31, 32].

Further influences could be caused by differences in body mass index (BMI), hormonal factors, and other sex-related factors. Although BMI was also not assessed in the present patient cohort, we expect a similar distribution in our patient population as BMI distribution was similar between the sexes in the general German population (ages 18–40: 25.4 for males vs. 23.4 for females; ages 40–65: 27.3 for males vs. 25.3 for females; Statistisches Bundesamt, https://www.destatis.de). Regarding hormonal factors, it is for example known that estrogens may provide parallel neuroprotective benefits on the stability of the BBB/BCSFB by preventing tight-junction breakdown through the up-regulation of anti-inflammatory annexin 1 (ANXA1) expression and by limiting lymphocyte migration through the modulation of the endothelial intercellular adhesion molecule 1 (ICAM-1) [33], which could indeed contribute to a better functioning of the BBB/BCSFB in females. Other hypothetical causes for the observed sex differences could include a sex-specific expression of secretion molecules, tight junctions or endothelial cells, but also sex-differences in the number of patients with consumption of alcohol or (illegal) drugs in the past.

### Clinical consequences

Irrespective of the cause, the Q_Alb_ findings should be corrected for sex ratio in future CSF studies in psychiatric and neurological patients. In addition to the established age-dependent correction, the present results suggest adapting or stratifying the reference values of Q_Alb_ for sex. If our considerations regarding body height are confirmed in future studies, a correction based on body size or spine length would be preferable. Nevertheless, it should be recognized that even with corrected Q_Alb_ a relevant subgroup with BBB/BCSFB dysfunction will persist, which cannot be explained by the bias and thus may relate to yet undefined brain pathologies as suggested from findings of abnormal Q_Alb_ in various psychiatric and neurological disorders [20, 34–39]. Additionally, it should be noted that a number of other CSF abnormalities have been reported in psychiatric patient cohorts [20, 38, 40–46]. Progress in this research area for psychiatry will require new and more sensitive methods of CSF analysis and an increased understanding of the complex interactions of biochemical and mechanistic influences on the dynamics of CSF contents under normal and pathological conditions.

### Limitations

The limitation of the present study is its retrospective, open, and uncontrolled design. Patients with affective syndromes received an LP only in selected cases because the diagnostic approach has evolved through the years [20, 43–45]. Therefore, the sex differences are not representative of all patients with affective syndromes. Due to the open design, patients with neurological comorbidity were also included. Therefore, BBB/BCSFB dysfunctions could, theoretically, also be caused by pathophysiology related to comorbid conditions. However, the Q_Alb_ values remained significantly different between sexes even when restricting analyses to patients without neurological comorbidity. Examinations of large, healthy control groups are lacking so far, so the present findings cannot be generalized to neurologically and/or mentally healthy populations because LPs of a large group of healthy volunteers is difficult to justify on ethical grounds. In contrast to previous studies [18, 19], the authors examined a more homogenous, well-characterized group of psychiatric patients. Another limitation is that the Q_Alb_ levels were not corrected for blood admixture and this could have influenced the results; however, this effect would apply equally to female and male patients. Unfortunately, the influences of other possible contributing factors, such as body weight, alcohol/nicotine consumption, or diabetes mellitus on BBB/BCSFB function, could not be analyzed in the present study, and body height was only estimated based on the mean heights of the current German population. In addition, exact measures of spine length or suboccipital-to-puncture-site distance for our physiological explanation of the bias were not available. A correction for the potential influence of psychotropic drugs was not performed, because equal numbers of male and female patients had been treated.

## Conclusions

The current study reveals a significant sex difference in Q_Alb_ levels, with higher levels in males than in females. The present results confirm and extend previous findings of a systematic sex difference in Q_Alb_, which exists not only in neurological patient cohorts but also in psychiatric patients, independent of diagnosis. Irrespective of the underlying pathophysiology, which remains to be elucidated, the present findings suggest the need for an extended correction mode of Q_Alb_ reference values in the future.

## Data Availability

All necessary information is displayed descriptively in the results section.
